# Diagnostic importance of AgNOR pleomorphism in cervical carcinogenesis

**DOI:** 10.3332/ecancer.2013.287

**Published:** 2013-01-09

**Authors:** AN Srivastava, S Srivastava, C Bansal, JS Misra

**Affiliations:** 1 Pathology Department, Era’s Medical College and Hospital, Lucknow, India; 2 Genetics Department, Sanjay Ghandi Postgraduate Institute of Medical Sciences (SGPGIMS), Lucknow, India

**Keywords:** single and pleomorphic AgNOR dots, cervical precancer, cancer

## Abstract

**Objective:**

Nucleolar organizer region (NOR) associated proteins are argyrophilic and visualized by silver stains. AgNOR pleomorphic dots increase in cancer and most researchers have done a common count of single dots. Pleomorphic dots are few and perhaps indicate a more severe prognosis. The present study was aimed at investigating the relative preponderance and diagnostic value of both pleomorphic and single AgNOR dots in cervical carcinogenesis.

**Study design:**

Silver nitrate staining was performed in 50 cervical smears each of cytologically diagnosed normal, inflammatory, low grade squamous intraepithelial lesion (LSIL), High-grade squamous intraepithelial lesion (HSIL), and squamous cell carcinoma cases registered at C.S.M. Medical University, Lucknow, India.

**Results:**

The accumulated data revealed a positive and significant correlation of cell counts of both pleomorphic (*r* = 0.94; *p* < 0.01) and single dots (*r* = 0.95; *p* < 0.01) with disease severity. The rate of increase in cell counts of pleomorphic dots (β = 2.61) was 1.1 times higher than the rate of increase in cell counts of single dots (β = 2.29).

**Conclusion:**

This study indicates the diagnostic potential of pleomorphic dots in the process of cervical carcinogenesis. The number of pleomorphic dots also varies significantly in different types of SIL, which may help in discriminating precancerous lesions of the cervix.

## Introduction

AgNOR proteins or AgNORs are chromosomal loops of DNA involved in ribosomal synthesis, which are stained with silver methods as black dots in the nuclei. Their size and number reflect the nucleolar and cell proliferative activity of tumours. AgNOR number and distribution in the nucleus (configuration) are useful in tumour detection and prognosis.

Currently, various studies are being conducted to explore the possibility of finding out the tumour marker potential of AgNOR dots. This has been necessitated because the technique is very easy, inexpensive, less time consuming, and provides accurate information on neoplastic development. Previously, studies were aimed at counting the entire AgNOR dots and correlating their quantity with different stages of premalignant and malignant transformation, and there were some studies carried out in cervical smears in this direction [1–4]. We have also previously reported on AgNOR dots with the severity of pathological lesions of cervix and also their role in discriminating different grades of SIL [5].

Since there are recent reports that polymorphism of AgNOR is more precisely related with proliferative activity with several pathological conditions [6–9], we thought it interesting to re-evaluate our material from cervical lesions including precancer and cancer for differentiating the pleomorphic dots from the single ones and measuring them quantitatively. This has become imperative in view of the findings of Alarcón-Romero *et al *that the pleomorphic dots are related to the HPV infection [10]. In the present study, AgNOR dots, both pleomorphic and single, have been counted in cervical smears under different pathological conditions of the uterine cervix, and the data has been analysed to discover the relation between the quantity of the two types of dots with the progression of the disease.

## Materials and methods

Fifty cases each of cytologically diagnosed normal, inflammatory, different grades of squamous intraepithelial lesions (SIL); LSIL, HSIL and squamous cell carcinoma were registered from the ongoing cytological screening program at the Out Patient Department of Queen Mary Hospital of C.S.M. Medical University after approval by the local institutional review board in Lucknow, India. The cervical smears of these cases were destained and stained with the silver staining method for the AgNOR proteins, without being counterstained. The smears were examined under 100 x oil immersion for AgNOR staining, and both pleomorphic and single small dots were counted. The count was done in 100 squamous cells in the normal and inflammatory smears; however, since it is difficult to get 100 dysplastic or malignant cells in the smears, the counts were made in approximately 15-20 cells showing pathology. For counting AgNOR dots, the criteria suggested by Crocker *et al *have been followed [11]. As all the argyrophilic dots visible in the nucleus are nucleolar organizer regions (NOR), all the silver-stained extra and intranucleolar structures were counted. All the pleomorphic and single small dots were counted individually, and the mean number of AgNORs, both pleomorphic and single, per nucleus was calculated for each smear.

## Statistical analysis

The groups were compared using one-way repeated measures analysis of variance (ANOVA) followed by the Newman-Keuls post-hoc test. The homogeneity of variance was tested by Hartley *F*-max, Cochran *C*, and Bartlett chi-square (*x*^2^) tests. The relative association between AgNOR counts (pleomorphic and single) and disease severity was assessed using the Pearson correlation method; assessing the normal groups as 1, inflammations as 2, LSIL as 3, HSIL as 4, and malignant as 5. A relative change (increase/decrease) of each AgNOR counts with disease severity was also assessed by β coefficient (slope) of simple linear regression analysis considering the counts as dependent variables and disease severity the independent variable. All the analyses were performed on square root transformed data. For interpretation of the results, the summarization (mean + SE) was done on actual data. A two-tailed (α = 2) probability *p *< 0.05 was considered to be statistically significant.

## Results

**Figure 1:**
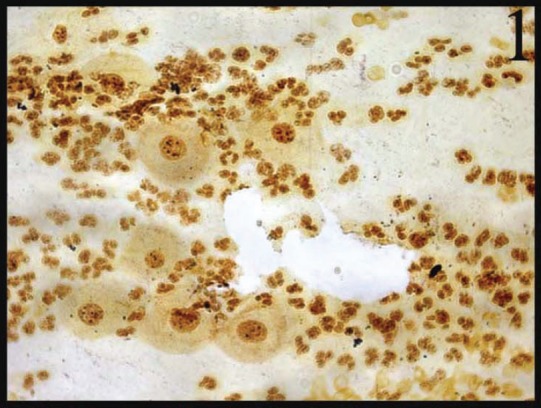
Inflammatory cervical smear shows 2–3 pleomorphic dots (AgNOR × 100)

**Figure 2:**
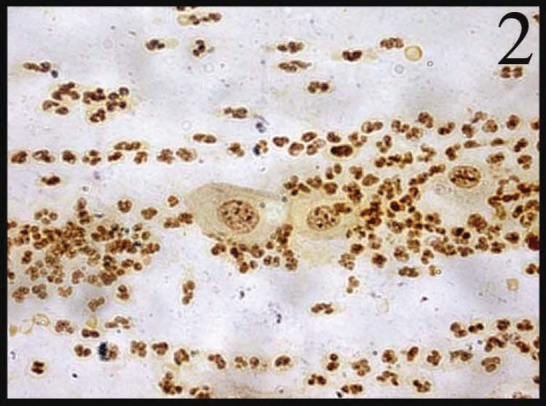
LSIL: cervical smear shows 3–4 pleomorphic dots (AgNOR × 100)

**Figure 3:**
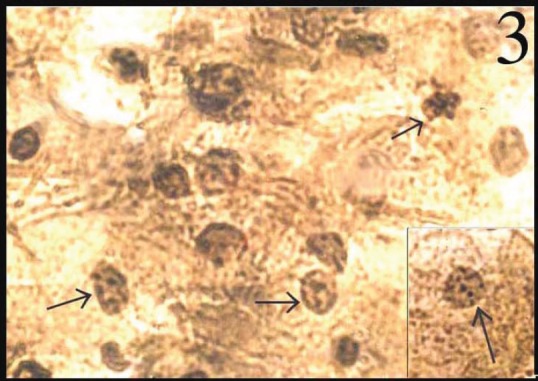
HSIL: nuclei in the cervical smear contain 5–6 pleomorphic dots (AgNOR × 400)

**Figure 4:**
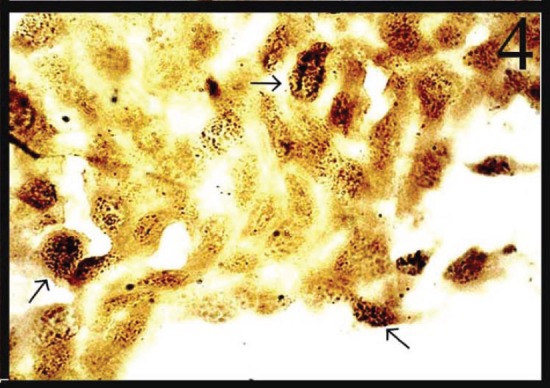
Squamous cell carcinoma cells dispersed in sheet exhibiting 8–10 pleomorphic dots (AgNOR × 100)

In normal smears, the number of total AgNOR dots ranged from 1 to 2, and in 41.8% of cases the dots were pleomorphic. In the inflammatory smears, the number of AgNOR dots varied from 2 to 3 (Figure 1), and the percentage of cases showing pleomorphic dots was 45.3%. In LSIL, total AgNOR dots were found to be 3–4 (Figure 2), and the percentage of cases showing pleomorphic dots was 48.3%. In the case of HSIL, AgNOR dots varied from 5 to 7 (Figure 3), and the percentage of cases showing pleomorphic dots was 56.7%. In the frank cancer cases, the total AgNOR dots were found to be 8–10 (Figure 4) and in 62.9% of cases, the dots were pleomorphic.

The pleomorphic and single AgNOR cell counts (counts/100 cells) consisting of five groups, namely: normal; inflammatory; two grades of SIL and squamous cell carcinoma are summarized in Table 1. The mean cell counts of both pleomorphic and single dots showed an increasing trend with disease severity, and the increase was more evident in the single dots rather than in the pleomorphic dots (Table 1). The mean cell counts of single dots in the normal, inflammatory, LSIL, HSIL, and malignant smears were 1.7 (40.1%), 1.9 (48.2%), 1.3 (21.0%), 1.25 (17.15%), and 1.2 (13.5%) fold higher than the respective mean cell counts of pleomorphic dots.

The variances of cell counts of both pleomorphic and single dots in all groups were found to be homogeneous (*p *> 0.05). The ANOVA revealed significantly different cell counts within (*F *= 68.19; *p *< 0.01) and between (*F *= 788.63; *p *< 0.01) the groups. The interaction of the groups was also found to be significant (*F *= 8.31; *p *< 0.01). Comparing mean cell counts within the groups (pleomorphic versus single), the cell counts of single in all the groups were found to be significantly (*p *< 0.05 or *p *< 0.01) different and higher than the pleomorphic (Table 1). Similarly, comparing the mean cell counts between the groups, the counts of pleomorphic dots in all groups differed significantly (*p *< 0.05 or *p *< 0.01) except between the normal and inflammation groups (Table 1). However, the counts of single dots in all groups differed significantly (*p *< 0.05 or *p *< 0.01) (Table 1).

The correlation of cell counts of both pleomorphic (*r *= 0.94; *p *< 0.01) and single dots (*r *= 0.95; *p *< 0.01) with disease severity was positive and significant. The correlation of cell counts between pleomorphic and single dots was also found to be positive and significant (*r *= 0.89; *p *< 0.01). But with severity, the rate of increase in cell counts of pleomorphic dots (β = 2.61) was 1.1 times higher than the rate of increase in cell counts of single dots (β = 2.29). 

## Discussion

The present study revealed AgNOR pleomorphism increasing with the severity of cytopathological lesions of the cervix. The difference was found to be more pronounced with pleomorphic dots rather than with single dots. Hence, pleomorphic dots can be very useful in establishing the prognosis of the disease and may play a crucial role in discriminating different grades of SIL and ascertaining the diagnosis of borderline cases.

Many investigators have also reported the usefulness of AgNOR pleomorphoism in assessing the progression of neoplasia in the cervix. They have found the pleomorphism of AgNOR increasing with progression of histopathological lesions, and the highest number was seen in squamous cell carcinoma [8–10]. The study assumes importance with the report of Alarcón-Romero *et al *that a significant difference was found with different types of AgNOR dots with infection of high-risk HPV types [10]. This indicated that during the progression of the lesions, HPV induces the progressive increase in the cellular proliferation. It appears that the presence of atypical AgNOR dots area product of cellular alterations that are clearly related not only to viral integration with the infected cell’s DNA but also to the progression of the lesion. They have also found that a compound study of viral parameters such as HPV types along with cellular proliferative markers such as polymorphism of the AgNORs can be useful as prognostic factors to estimate the progression of the premalignant lesions to squamous cell carcinoma.

As all the previous studies as well as the present study have established that there is a relationship between AgNOR pleomorphism and neoplastic development in the uterine cervix and there is speculation that a correlation exists between AgNOR pleomorphism and HPV infection, our suggestion is that AgNOR counts can replace HPV-DNA testing to discover high-risk SIL cases for follow-up for ascertaining the progression of the disease. This arrangement can be very useful in developing countries like India where Government-sponsored Cancer Control Programs have not been able to afford expensive HPV-DNA testing due to the financial expense. Pap smear examination supplemented with AgNOR pleomorphism estimation in SIL cases, is relatively less expensive and less time consuming, appears to be the most appropriate and feasible mode for control of the menace of cervical cancer in these countries.

## Figures and Tables

**Table 1.** AgNOR cell count (counts/100 cells) summary (Mean ± SE) of pleomorphic and single dots in five groups of subjects compared with normal uterine cervical smears.

Groups	Pleomorphic	Single
Normal	56.94 ± 2.81	95.00 ± 4.09**
Inflammation	71.02 ± 2.64	^a^ 137.14 ± 3.72**
LSIL	^a^^b^ 48.60 ± 4.81	^a^^b^ 188.14 ± 4.46*
HSIL	^a^^b^^c^ 264.75 ± 7.23	^a^^b^^c^ 325.75 ± 6.24**
Malignant	^a^^b^^c^^d^ 383.33 ± 11.84	^a^^b^^c^^d^ 443.33 ± 11.20*

Characteristics in *superscript *at right of ‘Single’ showed comparison within groups (i.e. Pleomorphic vs. Single dots)

Characters in *superscript *at left of both ‘Pleomorphic and Single’ showed comparison between the groups and were significant at level *p *< 0.05 or *p *< 0.01.

a Comparison with ‘normal’

b Comparison with ‘inflammation’

c Comparison with ‘LSIL’

d Comparison with ‘HSIL’

* *p *< 0.05,

** *p *< 0.01
